# Neuroendocrine carcinoma of the esophagus: Clinicopathological and immunohistochemical features of 14 cases

**DOI:** 10.1371/journal.pone.0173501

**Published:** 2017-03-13

**Authors:** Akinori Egashira, Masaru Morita, Reiko Kumagai, Ken-ichi Taguchi, Masanobu Ueda, Shohei Yamaguchi, Manabu Yamamoto, Kazuhito Minami, Yasuharu Ikeda, Yasushi Toh

**Affiliations:** 1 Department of Gastroenterological Surgery, National Kyushu Cancer Center, Fukuoka, Japan; 2 Department of Cancer Pathology, National Kyushu Cancer Center, Fukuoka, Japan; 3 Department of Radiology, National Kyushu Cancer Center, Fukuoka, Japan; Seconda Universita degli Studi di Napoli, ITALY

## Abstract

**Background:**

Neuroendocrine carcinoma (NEC) of the esophagus is a rare and highly aggressive disease but the biological features are poorly understood. The objective of this study was to analyze the clinicopathological and immunohistochemical features of NEC of the esophagus.

**Methods:**

Fourteen patients diagnosed with NEC of the esophagus from 1998 to 2013 were included in this study. Clinicopathologic features, therapeutic outcomes and immunohistochemical results were analyzed.

**Results:**

Eleven out of 14 cases showed protruding or localized type with or without ulceration. Only three patients were negative for both lymph node and organ metastasis and seven cases were positive for metastases to distant organs and/or distant lymph nodes. Of the six patients with limited disease (LD), three patients were treated by surgery. Three patients with LD and seven patients with extensive disease (ED) were initially treated with chemotherapy, except for one who underwent concurrent chemo-radiotherapy due to passage disturbance. The median survival of patients with LD was 8.5 months, whereas that of patients with LD was 17 months. Among the 14 cases, 12 cases (83.3%), 13 cases (91.7%) and 12 cases (83.3%) showed positive immunostaining for choromogranin A, synaptophysin and CD56, respectively. Nine of 14 cases (64.2%) presented positive staining for c-kit and most (8/9, 88%) simultaneously showed p53 protein abnormality. Two cases were negative for c-kit and p53, and positive for CK20.

**Conclusion:**

Consistent with previous reports, the prognosis of NEC of esophagus is dismal. From the results of immunohistochemical study, NEC of esophagus might be divided into two categories due to the staining positivity of c-kit and p53. This study provides new insight into the biology of NEC of the esophagus.

## Introduction

Neuroendocrine carcinoma (NEC) is a relatively rare disease with a reported incidence between 0.4% and 2% among all malignancies of the esophagus [1–3]. NEC is categorized into two morphological types: small cell type and large cell type. The former is more frequent (approximately 90% of total cases) and most cases formerly recognized as small cell carcinoma or oat cell carcinoma of the esophagus were included [4].

The WHO definition for NEC includes positive endocrine marker such as choromogranin A, synaptophysin and CD56. A Ki67 or mitotic index of 20% or more is also necessary for diagnosing NEC, the tumors with less than 20% Ki 67 positivity are diagnose as neuroendocrine tumors [5]. Macroscopically, NEC of the gastrointestinal tract presents relatively prominent features including submucosal growth, usually covered by normal epithelium with or without ulcerous lesion in the center. Microscopically, the features of neuroendocrine cells are formation of nested and trabecular growth with peripheral palisading and rosette formation in the tumors. High frequency of venous invasion, lymphatic invasion and perineural invasion are also seen [1].

The prognosis of NEC of the esophagus is poor, as the tumor is often at advanced disease at diagnosis. The therapeutic strategy for NEC of the esophagus has not been well defined due to the small number of cases reported in the literature thus far [6, 7]. NEC can be categorized into two groups: limited disease (LD) and extended disease (ED) [8]. For ED, chemotherapy is the predominant treatment strategy, and radiotherapy is also applied for some cases. For LD, the therapeutic strategy is more complicated. In some studies, long-term survivors are treated with surgical resection (esophagectomy and extended lymph node dissection) with or without adjuvant chemotherapy [9, 10]. However, even for LD, multi-modality treatments such as surgery followed by adjuvant chemo-(radio)-therapy or neo-adjuvant chemotherapy followed by surgery are commonly recommended [6]. Although the role of surgery is controversial, the co-existence of squamous cell carcinoma with NEC makes it difficult for complete response by chemotherapy which is effective for NEC but not for squamous cell carcinoma [1]. From this point of view, multimodal treatment including surgical resection could be considered.

Here we present 14 cases of NEC of the esophagus and discuss the diagnostic and therapeutic points as well as biological features.

## Materials and methods

### Ethics statement

This research have been approved by the Institutional Review Board in National Kyushu Cancer Center.

### Biopsy samples

Fourteen biopsy samples were collected from 1998 to 2013 at the National Kyushu Cancer Center Hospital. Five samples were primarily diagnosed as NEC. Nine samples were initially diagnosed as small cell carcinoma or undifferentiated carcinoma and examined for the neuroendocrine markers (choromogranin A, synaptophysin and CD56). Tumors positive for one of these markers were eventually diagnosed as NEC.

### Patients

Fourteen patients diagnosed with NEC of the esophagus were reviewed by their clinical medical records. Written informed consent was collected from each patient. Since the specific staging system for NEC of the esophagus is lacking, both staging classifications of the AJCC for esophageal carcinoma (seventh edition) [11] and the Veterans’ Administration Lung Study Group (VALSG) for primary pulmonary small cell carcinoma [8] were applied for clinical staging. For the latter, patients were categorized into two groups according to the extent of the disease. Limited disease (LD) was defined as tumors confined within a localized anatomic region with or without regional lymph node involvement, whereas extensive disease (ED) was defined as tumors outside loco-regional boundaries.

### Immunohistochemistry

Immunohistochemical stainings were carried out using the BOND-III automated immunohistochemical stainer (Leica Biosystems, Nussloch, Germany). All antibodies used in this study were commercially purchased: choromogranin A (IS50230, dilution 1:1000, Dako, Glostrup, Denmark), synaptophysin (PA0299 Bond Ready-To-Use Primary antibody, Leica Biosysytems, Nussloch, Germany) and CD56 (CD56-1B6-R-7 Bond Ready-To-Use Primary antibody, Leica Biosysytems, Nussloch, Germany), Ki67 (M7240, dilution 1:100, Dako, Glostrup, Denmark), c-kit (A4502, dilution 1:100, Dako, Glostrup, Denmark), p53 (N1581, dilution 1:10, Dako, Glostrup, Denmark), p63 (413751, dilution 1:5, Nichirei, Tokyo, Japan), CK5/6 (M7237, dilution 1:250, Dako, Glostrup, Denmark) and CK20 (413491, dilution 1:1000, Nichirei, Tokyo, Japan). Appropriate positive and negative controls were implemented in each reaction. The tumor samples were regarded as positive when more than 10% of nuclei were stained for p53 as previously reported [12]. The positivity of c-kit was evaluated by the immunoreactivity of cytoplasm in the tumor cells as previously reported [1]. Each immunostaining was reviewed and independently scored by two pathologists independently (K. T. and R. K.).

### Survival analysis

Survival was estimated by Kaplan-Meier method using JMP11 software (SAS Institute Inc., Cary, NC).

## Results

### Clinicopathologic features of patients NEC of esophagus

**Fig 1:**
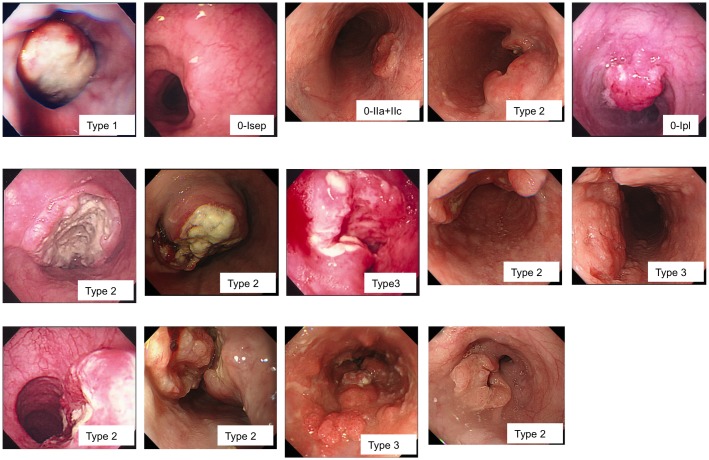
Endoscopic findings of 14 cases with neuroendocrine carcinoma of esophagus. Eleven out of 14 cases showed protruding or localized type with or without ulceration in the center.

Between 1998 and 2013, 14 patients were diagnosed with NEC of the esophagus at the National Kyushu Cancer Center Hospital. Clinicopathologic features of these patients are listed in Table 1. The median age of the patients was 69 years (ranging from 49 to 79 years), and the female-to-male ratio was 1:3.7. Regarding the location of the tumors, 3, 5 and 6 tumors were located at the upper, middle, and lower third of the esophagus, respectively. Eleven out of 14 cases showed protruding or localized type with or without ulceration in the center (Fig 1). For clinical stage according to the extent of tumor, 6 of 14 patients were LD. By UICC staging system [13], 3 patients were diagnosed as clinically stage IA, 1 patient was stage IIB, 3 patients were stage IIIA/C, 7 patients were Stage IV.

**Table 1:** Clinicopathologic features of neuroendocrine carcinoma of esophagus.

Case	Age	Sex	Location	Type	Length (cm)	Depth	Lymph node	Distant organs	cStage
1	75	F	L	1	3.5	1b	0	none	IA
2	71	M	M	0-Isep	1.2	1b	0	none	IA
3	72	F	M	0-IIa+IIc	1.8	1b	0	none	IA
4	73	M	M	2	6	2	1	none	IIB
5	78	M	U	0-Ipl	1.6	3	1	none	IIIA
6	76	M	L	2	4	3	1	none	IIIA
7	49	M	L	2	4	4a	2	none	IIIC
8	58	M	U	3	12.5	2	M1^a^	none	IV
9	59	F	L	2	3	2	M1^b^	none	IV
10	71	M	U	3	5	3	M1^a^	liver	IV
11	75	M	M	2	4.7	3	0	liver	IV
12	75	M	L	2	5	3	3	liver, bone	IV
13	64	M	L	3	13	3	3	liver	IV
14	79	M	M	2	6	3	M1†	liver	IV

location: U, upper; M, middle; L, lower

^a^ supraclavicular LN,

^b^ abdominal paraaortic LN

Elevated values of tumor markers were detected in 35.7% (5/14) for CEA, 7.1% (1/14) for SCC, 44.4% (4/9) for NSE and 50% (3/6) for Pro-GRP (Table 2).

**Table 2:** Clinicopathologic features of neuroendocrine carcinoma of esophagus.

Case	CEA	SCC	NSE	Pro-GRP	Treatment	Period (Months)	Status
1	1.4	0.4			Surg	>70	alive
2	1.5	0.6	6.4 (<10)	29.5 (<46)			
3	**5.3**	0.4	15.7 (<16.3)		Surg	7	died
4	1.1	0.7	12.8 (<16.3)	**267** (<46)	Surg, CTx	12	alive
5	4.6	0.4			CTx	17	died
6	2.6	0.8	**11** (<10)	**171** (<46)	CTx, RTx	10	died
7	1.6	0	8.6 (<10)	18.3 (<70)	CTx, RTx	17	died
8	1.2	**2.3**			CRTx	8	died
9	**5.9**	1.4			CTx		
10	**19.6**	1.1	**130** (<10)	**1670** (<46)	CTx	9	died
11	**7.8**	0.8	**28** (<10)		CTx, RTx	13	died
12	2.4	0.7			CTx	14	died
13	2.3	1.6	12.8 (<16.3)	25.8 (<46)	CTx, RTx	6	died
14	**13.9**	1	**17.4** (<16.3)		CTx	4	died

Surg; Surgery, CTx; Chemotherapy, RTx; Radiotherapy, CRTx; Chemoradiotherapy

### Treatments and prognoses

Treatments and prognoses are also listed in Table 2. The choice of initial treatment depends on the extent of disease. Of the six patients with LD, three patients were treated by surgery. Of these three, one patient (case 4) received adjuvant chemotherapy of cisplatin/etoposide and the remaining two received no adjuvant therapy. Case 1 was pathologically stage I, and case 3 was pathologically stage II disease. However, we could not perform adjuvant chemotherapy for case 3 because of liver dysfunction. The patient who underwent surgery alone (case 1) survived over 70 months without recurrent disease. One patient with LD underwent initial chemotherapy of cisplatin/irinotecan (case 6) and another patient did chemotherapy by fluoropyrimidine/irinotecan (case 5) according to the patient’s request. One of two patients underwent subsequent radiotherapy due to passage disturbance by tumor progression. One patient with LD refused to undergo any treatment (case 7).

Of the eight patients with ED, initial chemotherapy was applied for all except for one who underwent concurrent chemo-radiotherapy due to passage disturbance. All eight patients received chemotherapy containing platinum compound (either cisplatin or carboplatin) and irinotecan or etoposide or 5-fluorouracil. One patient with ED could not be followed because of the hospital transfer.

**Fig 2:**
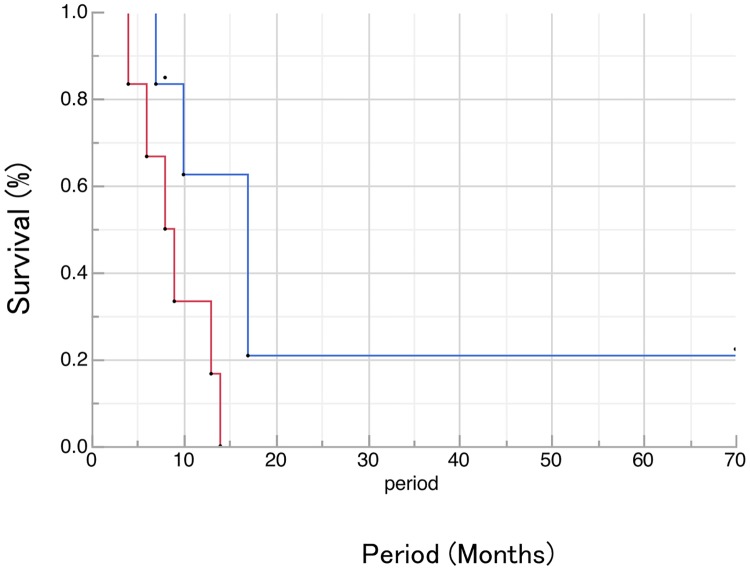
Overall survival in patients of neuroendocrine carcinoma of esophagus with LD or ED. The MST of patients with ED was 8.5 months, whereas the MST of patients with LD was 17 months.

The median survival time (MST) of patients with ED was 8.5 months, whereas the MST of patients with LD was 17 months (Fig 2).

### Immunostaining of neuroendocrine markers (choromogranin A, synaptophysin and CD56) and other markers

According to the WHO recommendation, we examined three neuroendocrine markers (choromogranin A, synaptophysin and CD56) to screen NEC of the esophagus. Among 14 cases, 12 cases (83.3%), 13 cases (91.7%) and 12 cases (83.3%) showed positive immunostaining for choromogranin A, synaptophysin and CD56, respectively (Table 3). The average index of Ki67 index was 63±26%, ranging from 21% to 96%.

**Table 3:** Imuunostaining of neuroendocrine markers (choromogranin A, synaptophysin and CD56) and other markers.

Case	Chromo-granin A	Synapto-physin	CD56	Ki67 (%)	c-kit	p53	p63	CK5/6	CK20
1	(-)	(+)	(+)	67	(±)	(+)	(-)	(-)	(-)
2	(+)	(+)	(-)	21	(-)	(-)	(-)	(-)	(+)
3	(+)	(+)	(+)	94	(+)	(+)	(-)	(-)	(-)
4	(+)	(+)	(-)	96	(+)	(+)	(-)	(-)	(-)
5	(+)	(+)	(+)	34	(+)	(+)	(-)	(-)	(-)
6	(+)	(+)	(+)	71	(±)	(-)	(-)	(-)	(-)
7	(+)	(+)	(+)	77	(-)	(-)	(-)	(-)	(-)
8	(+)	(+)	(+)	50	(+)	(+)	(-)	(-)	(-)
9	(+)	(-)	(+)	45	(-)	(±)	(-)	(-)	(-)
10	(+)	(+)	(+)	73	(+)	(+)	(-)	(-)	(-)
11	(+)	(+)	(+)	24	(+)	(+)	(-)	(-)	(-)
12	(-)	(+)	(+)	52	(-)	(-)	(-)	(-)	(+)
13	(+)	(+)	(+)	82	(-)	(+)	(-)	(+)	(-)
14	(+)	(+)	(+)	93	(+)	(+)	(-)	(-)	(-)

**Fig 3:**
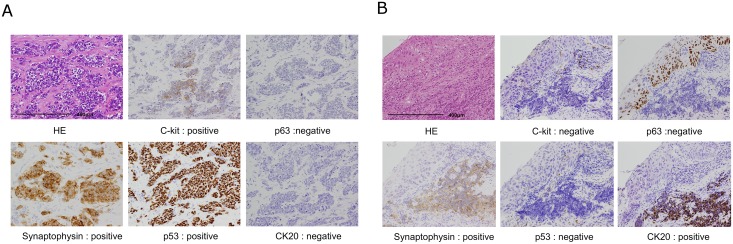
Immunohistochemical pictures of neuroendocrine carcinoma of esophagus. A) Case 3 shows positive staining for c-kit and p53 protein. B) Case 13 shows positive staining for CK20, and negative staining for both c-kit and p53 immunostainings.

To characterize these tumors more precisely, we analyzed another protein markers such as p53, c-kit, p63, CK5/6 and CK20. p53 has been reported to be important for carcinogenesis of esophagus [12]. Some of NEC cells has been showed positive c-kit expression by immunohistochemical analysis [14], whereas c-kit has important role for gastrointestinal stromal tumor. p63, CK5/6 and CK20 were also examined to examine the origin of cells. Nine of 14 cases (64.2%) presented positive staining for c-kit and most of these cases (8/9, 88%) simultaneously showed p53 protein abnormality. Two cases were positive for CK20 and negative for both c-kit and p53 immunostaining. The CK20 was regarded to be the marker for Merkel cells which found to be numerous in the middle esophagus and thought to have neuroendocrine features[15]. Representative pictures of immunostaining for Case 3 and Case 13 are shown in Fig 3. Based on the immunostaining results, we were able to divide the staining patterns into two classes: one is positive for c-kit and p53, and negative for CK20; and the other is negative for c-kit and p53, and positive for CK20. As we showed the representative pictures in Fig 3a, p53 and neuroendocrine markers, such as synaptophysin, were relatively stained uniformly. On the other hand, the immunoreactivity of c-kit was irregular and it might be explained by the heterogeneity of tumors [1]. Previous studies reported the co-existence of squamous cell carcinoma or adenocarcinoma with NEC of the esophagus. Immunostaining showed that the squamous cell carcinoma component is positive for p63 and negative for CK20, and the, the NEC component is negative for p63 and positive for CK20 (Fig 3b).

## Discussion

NEC was formerly regarded as small cell carcinoma and the first description of small cell carcinoma of the esophagus was reported in 1952 by McKeown [4]. The incidence of NEC is relatively low, ranging between 0.4% and 2% in all malignancies of the esophagus [1–3]. The chance to encounter this malignancy in clinical setting is also increasing, probably due to the widened publicity of the disease from the WHO definition. In fact, in our series of 14 cases collected from 1998 to 2013, half of the cases (7 of 14) were diagnosed after 2010.

Characteristic macroscopic features of NEC include submucosal growth, which might be helpful to diagnose this disease. Heterozygosity is also frequently observed in neuroendocrine NEC of the esophagus as a pathologic feature and co-existence of squamous cell carcinoma and/or adenocarcinoma are also often observed. Huang reported that over 80% of NEC of the esophagus has synchronous squamous neoplasm including tumor in situ [1]. Case 13 showed an initial diagnosis of squamous cell carcinoma with concomitant liver tumor but pathological examination from synchronous liver tumor showed the features of NEC and eventual re-biopsy of samples from the esophageal tumor showed both neuroendocrine and squamous neoplasm in individual sites from esophagus.

The cellular origin of NEC of the esophagus remains unknown, but two possible candidates include Merkel cells and stem cells. Considering this potential origin of NEC cells, it is interesting that NEC cells show positive c-kit expression by immunohistochemical analysis [16]. The positive expression for c-kit protein is frequently observed in the gastrointestinal stromal tumor (GIST) that originates from Cajal cells in the gastrointestinal wall. There might be some association between Cajal cells and the cells from which NEC originate. In our series of histological analyses, 9 of 14 cases (64.2%) presented positive staining for c-kit and most (8/9, 88%) simultaneously showed p53 protein abnormality. Similar to squamous cell carcinoma of the esophagus in which the abnormality of p53 plays an important role in carcinogenesis [12], the abnormality of p53 might play some role in the carcinogenesis or progression for NEC of the esophagus. In pulmonary neuroendocrine tumors, protein abnormality, loss of heterozygosity and gene mutation were observed and the incidence of these abnormalities progressively increased with increasing severity of tumor type [17].

Among the tumors that showed negative staining for both c-kit and p53 proteins, there are two cases positive for CK20, which is a marker for Merkel cells. Huang reported a low frequency of CK20 positivity in NEC of the esophagus [1]. In our cohort, two cases with positive staining for CK20 showed negative staining for p53 abnormalities. There has been reported that p53 abnormality was also rare events in Merkel cell carcinoma of the skin [18]. There might be two different groups by means of originated cells of neuroendocrine carcinoma of the esophagus; one is positive for c-kit and p53, and the other is positive for CK20. To clarify the significance of immunohistochemical status on prognosis, we compared the survival according to the immunohistochemical status. There were no significant differences of prognosis according to the immunohistochemical status of p53 and/or c-kit, or others (CK20 and Ki67). Since these results might be due to the small cohort of our analysis, large scale of analysis should be performed to clarify the significance of immunohistochemical status on prognosis.

Although the therapeutic strategy for NEC of the esophagus is still not established, it is important to precisely diagnose it because its treatment might be completely different from that of esophageal squamous cell carcinoma and adenocarcinoma. Furthermore, the co-existence of squamous cell carcinoma or adenocarcinoma further complicates treatment. The standard therapy for NEC of the esophagus is not standardized even for the LD, the multi-modality treatments including surgery, chemotherapy and radiotherapy are recommended [6]. Although the role of surgery is controversial, the co-existence of squamous cell carcinoma with NEC makes complete response by chemotherapy difficult, as this is effective treatment for NEC but for squamous cell carcinoma. A randomized prospective study for determining the appropriate treatment for LD or ED esophageal neuroendocrine carcinoma is needed. Cisplatin/etoposide and cisplatin/irinotecan are two major regimens for esophageal NEC, and the effectiveness of amrubicin-based chemotherapy was also recently reported [19]. Furthermore, the possibility of a therapeutic candidate of c-kit-targeted therapy, such as Glivec or Sutent, should be examined for effectiveness for the NEC of the esophagus.

In conclusion, here we analyzed the clinicopathologic and immunohistochemical features of NEC of the esophagus. A larger study might be important for clarifying the molecular mechanisms underlying the NEC of the esophagus, and a prospective study should be performed for establishing the optimal treatment for this rare and dismal disease.

## Abbreviations

ED: extended disease
LD: limited disease
NEC: neuroendocrine carcinoma
